# Nurses' practices and their influencing factors in palliative care

**DOI:** 10.3389/fpubh.2023.1117923

**Published:** 2023-05-17

**Authors:** Yifan Xu, Shiwen Zhang, Jingrong Wang, Zhiqun Shu, Limei Jing, Jiangjiang He, Mengtian Liu, Tianshu Chu, Xiaohan Teng, Yanan Ma, Shuijing Li

**Affiliations:** ^1^School of Public Health, Shanghai University of Traditional Chinese Medicine, Shanghai, China; ^2^Disciplinary Planning Office, Shanghai Ninth People's Hospital, Shanghai Jiao Tong University School of Medicine, Shanghai, China; ^3^Centre for Specialty Strategy Research of China Hospital Development Institute, Shanghai Jiao Tong University, Shanghai, China; ^4^Department of Health Policy Research, Shanghai Health Development Research Center, Shanghai, China; ^5^School of Foreign Language Education, Jilin University, Jilin, China; ^6^Longhua Hospital, Shanghai University of Traditional Chinese Medicine, Shanghai, China; ^7^Department of Biostatistics and Epidemiology, School of Public Health, China Medical University, Shenyang, Liaoning, China; ^8^Department of Primary Health, Shanghai Municipal Health Commission, Shanghai, China

**Keywords:** nurse, practices, structural equation model, influencing factors, palliative care

## Abstract

**Background:**

In 2017, the Chinese government launched a pilot project in palliative care, in which Shanghai was a pioneer. Nurses play a key role in palliative care services as they are the main providers improving the quality of services for patients and their families. However, little is known about practices and influencing factors in the field of palliative care from a nursing perspective in China. This is an original empirical study that has meticulously analyzed the interrelationship and intensity between practices and other factors among nurses in the initial stage of palliative care in primary healthcare institutions in Shanghai, China.

**Methods:**

A descriptive-correlational study design was used to sample 2,829 eligible palliative care nurses by purposive sampling survey in 225 healthcare institutions in Shanghai, China. Descriptive analyses were performed using IBM SPSS 24.0 software. Structural equation modeling was applied to analyze the data by AMOS 20.0. Data were collected using the well-designed Knowledge, Attitudes, and Practices of Hospice Care (KAPHC) scale.

**Results:**

The final model showed a good model fit. Self-efficacy directly influenced practices (β = 0.506, *P* < 0.01) and indirectly influenced practices (β = 0.028, *P* < 0.01) through intention. Subjective norm directly influenced practices (β = 0.082, *P* < 0.01) and indirectly influenced practices (β = 0.030, *P* < 0.01) through intention. Intention (β = 0.152, *P* < 0.01) and knowledge (β = 0.068, *P* < 0.01) directly influenced practices. Perceived susceptibility (β = −0.027, *P* < 0.01), perceived benefits (β = −0.017, *P* < 0.01), and perceived barriers (β = −0.014, *P* < 0.01) indirectly influenced practices through intention.

**Conclusion:**

This study provided evidence of the associations of knowledge, perceived susceptibility, benefits, barriers, subjective norm, self-efficacy, intention, and practices among nurses concerning palliative care and interventions improving their actual work practices. Our findings revealed that self-efficacy, intention, and subjective norms greatly influenced practices. It is imperative to take interventions that focus precisely on self-efficacy, intention, and subjective norms to improve nurses' practices.

## Introduction

Palliative care is the active holistic care of individuals of all ages with serious health-related suffering due to severe illness and especially those near the end of their life (1). With two batches of national pilots launched in 2017 and 2019, China initially established a palliative care service system (2). In clinical palliative care practices, nurses play an active, and often lead role in managing the whole process of patients' disease diagnosis, treatment, and dying and death, as well as meeting their physical, spiritual, cultural, and religious needs during different periods (3). Previous studies have concluded that nurses should equip themselves with diversified psychological and compassionate care comprehensively, instead of previous simple physical symptom management (4, 5). In addition, previous studies have preceded that compared to physicians, nurses had more positive attitudes toward palliative care (6). Furthermore, in the initial stage of palliative care pilot work, there is a shortage of composition and proportion of a specialized team (7). The roles of social workers and psychological counselors in interdisciplinary groups were not fully involved (8, 9); therefore, nurses undertake multi-functional roles and diversified nursing such as communication. The behavioral practices of palliative care nurses directly affect the quality of palliative care services.

However, previous studies were mostly limited to small sample surveys of specialist nurses in one institution in China (10–12). There are scarce in-depth systematic studies focusing on the interrelationship between nurses' practices and large sample surveys on influencing factors. Therefore, based on the above current situation, it is important to comprehensively measure the behavioral profile of palliative care nurses and explore the facilitators of practice in China.

## Methods

### Study design and participants

This is a cross-sectional study based on purposive sampling. We investigated all health institutions registered with palliative care from November to December 2019, including hospitals, community health centers, and nursing homes covering the whole area of Shanghai. Moreover, 15 registered nurses were recruited from each institution ideally. If the number is < 15, all registered nurses were investigated. The participants' inclusion criteria were as follows: nurses (1) who were nurse practitioners and (2) who voluntarily agreed to participate in the anonymous survey.

Based on a strict logical structure, the questionnaire in Chinese was scientifically designed and electronic, and all key information is required to ensure that all returned questionnaires are valid. The anonymous questionnaire survey was conducted through SO JUMP, a professional online questionnaire survey platform used by a large number of companies and individuals. The QR link and code of the questionnaire were sent to the head nurses, who distributed the questionnaires to the nurses who met the inclusion criteria in their departments. They can fill in the questionnaire using mobile phones or computers. Finally, 2,829 nurses from 225 health institutions were investigated.

### Measures

This study employed a descriptive-correlational study by well-designed Knowledge, Attitudes, and Practices of Hospice Care (KAPHC) questionnaire demonstrating good validity and reliability (https://onlinelibrary.wiley.com/doi/10.1002/hpm.3074) (13, 14). The questionnaire contained five sections: demographic and relevant objective work-experience characteristics, knowledge (15 items), attitudes (24 items with four sub-concepts), confidence (11 items), and self-reported practices (11 items). Demographic and relevant objective work-experience characteristics included age (years), gender (male = 0, female = 1), educational level (junior middle school or less = 0, high school or vocational college = 1, and bachelor or above = 2), marital status (unmarried = 0, married = 1, and divorced or widowed = 2), nationality (minorities = 0, Han = 1), religious belief (none = 0, other = 1), professional title (none = 0, junior = 1, intermediate = 2, and senior = 3), the experience of death witness (no = 0, yes = 1), the willingness of providing palliative care (no = 0, yes = 1), and institution type (nurse home = 0, community health centers = 1, and hospital = 2). The Cronbach's α coefficient of knowledge, attitude, confidence, and practices scale was 0.686, 0.868, 0.960, and 0.971, respectively (13). Scores for each subcategory were calculated separately. Regarding the knowledge scale, a score of 1 was given for a correct answer and 0 for an incorrect or unknown answer. The overall score ranges from 0 to 15, with higher scores indicating better palliative care knowledge. Meanwhile, each item in the attitudes section was scored by a 5-point Likert scale (1 = totally disagree to 5 = totally agree) and higher scores indicate better attitudes. Self-efficacy was assessed by a confidence scale (1 = no confidence to 5 = extreme confidence). The overall score ranges from 11 to 55, with higher scores indicating greater self-efficacy. Practices were scored by a 5-point Likert scale (1 = never do to 5 = always do). The overall score ranges from 14 to 70, with higher scores indicating greater work practices.

### Model construction

**Figure 1:**
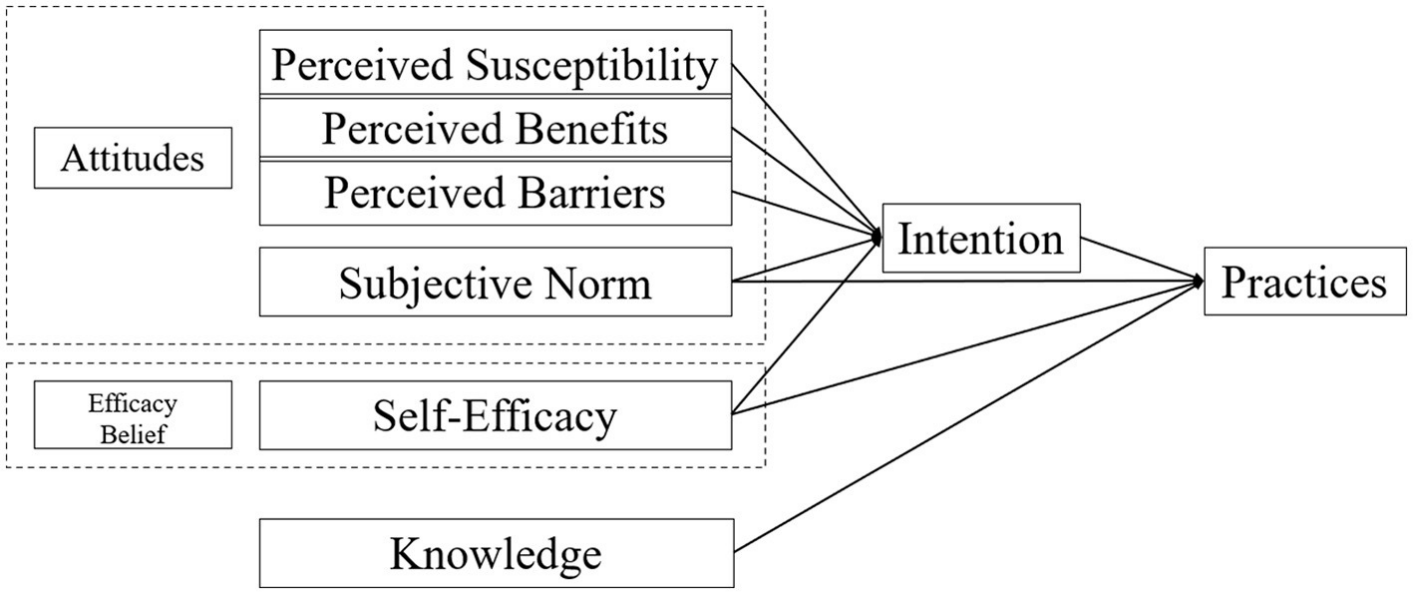
Hypothetical model for practices and their influencing factors among nurses concerning palliative care.

The theoretical framework in this study originates from three classical and widespread health and behavior theories: the Health Belief Model (HBM) (15), the Theory of Reasoned Action (TRA) (16), and the Integrated Behavior Theory (IBT) (17). According to the HBM theory, which is a model of health education that changes people's behavior by intervening in their psychological activities such as perceptions, attitudes and beliefs, perceived susceptibility, perceived benefits, perceived barriers, and self-efficacy influence behavior change. Based on the TRA, whether a person engages in behavior directly depends on their intention to act. In addition, subjective norm affects behavioral intention. IBT theory suggests that behavior is influenced by knowledge. Based on the theoretical components and dimensions mentioned in the above three theories and existing domestic and international research, knowledge, attitudes, self-efficacy, intention, and practices were incorporated into the hypothesis model (Figure 1) for confirmatory analysis. Behavioral intention is a fundamental intermediary factor, determined by self-efficacy and attitudes of individuals. Consequently, nurses' practices in palliative care may be affected by knowledge, attitudes, self-efficacy, and intention in our hypothetical model.

Therefore, in the current study, we verified the associations among knowledge, self-efficacy, subjective norm, perceived susceptibility, benefits, barriers, intention, and practices using the structural equation model. Based on the theoretical framework and literature review, we developed a model to identify the following hypotheses: (1) Intention directly influences practices. (2) Subjective norms and self-efficacy have direct and indirect effects on practices through intention. (3) Knowledge directly influences practices. (4) Perceived susceptibility, benefits, and barriers have indirect effects on practices through intention.

### Statistical methods

Descriptive data were generated for all variables. Statistical analyses were performed using commercial software IBM SPSS Statistics 24.0 and AMOS version 20.0 (IBM Corporation, Armonk, NY). The level of significance was set at a *p* < 0.05. Categorical data were described by frequencies and percentages, and continuous data by means of standard deviations (SD). Pearson's (r) correlation was performed to verify the relationship between all variables. 0 ≤ |r| < 0.3 means low correlation; 0.3 ≤ |r| < 0.8 means medium correlation; 0.8 ≤ |r| ≤ 1.0 means high correlation. A *P* < 0.05 was considered statistically significant. A structural equation model was used to test the associations between all variables. Path analysis was used to identify both direct and indirect relationships in the model. Standardized regression coefficients (β) and lower and upper bounds for β were reported for direct, indirect, and total effects. The model fit was assessed using the following model-fit indices: relative chi-square (χ^2^/df) < 3; goodness of fit index (GFI) > 0.900, adjusted goodness of fit index (AGFI) > 0.900, comparative fit index (CFI) > 0.900, normed fit index (NFI) > 0.900, incremental fit index (IFI) > 0.900, Tucker–Lewis index (TLI) > 0.900, and root mean squared error of approximation (RMSEA) < 0.06 (18).

## Results

### Participants' characteristics

A total of 2,829 nurses from 225 institutions across Shanghai's districts were enrolled. The demographic characteristics of the respondents are listed in Table 1. The nurses' work units included the following: community health centers (69.21%) were the most common units, followed by nursing homes (17.96%) and hospitals (12.83%). The mean age was 36.83 ± 9.35 years.

**Table 1:** Demographic characteristics of the respondents (*N* = 2,829).

	***N***	**%**
**Gender**		
Female	2,754	97.35
Male	75	2.65
**Age**		
≤ 30	1,244	43.97
31–50	1,487	52.56
≥51	98	3.46
**Educational degree**		
Bachelor or above	1,444	51.04
High school or vocational college	1,186	41.92
Junior middle school or less	199	7.03
**Marriage status**		
Unmarried	672	23.75
Married	2,082	73.59
Divorced or widowed	75	2.65
**Nationality**		
Han	2,744	97.00
Minorities	85	3.00
**Religious Belief**		
None	2,389	84.45
Other	440	15.55
**Professional title**		
Senior	48	1.70
Intermediate	963	34.04
Junior	1,570	55.50
None	248	8.77
**Experience of death witness**		
Yes	2,413	85.30
No	416	14.70
**Willingness of providing hospice and palliative care**		
Yes	1,752	61.93
No	1,077	38.07
***If yes, your main consideration is***		
It's a task from the superior.	269	15.35
It's my duty.	1,283	73.23
My religious belief.	43	2.45
It's charitable.	157	8.96
***If no, your main consideration is***		
It's stressful.	823	76.42
Low salary.	140	13.00
Unvalued.	32	2.97
Meaningless.	23	2.14
Blind-alley job.	59	5.48
**Institution type**		
Hospital	363	12.83
Community health centers	1,958	69.21
Nursing home	508	17.96

### Knowledge

The mean score of the knowledge scale was 8.39 ± 2.780; the response accuracy was 55.91%. Table 2 details the questions and scores.

**Table 2:** Respondents' knowledge of palliative care (*N* = 2,829).

**Items**	**Correct number (%)**	**Mean ±SD**
1. The provision of hospice care requires emotional detachment.	482 (17.04)	0.17 ± 0.376
2. Psychological, social, and spiritual problems are paramount to the hospice care team who give appropriate consultation and management.	2,468 (87.23)	0.87 ± 0.344
3. Three steps make up the WHO analgesic ladder.	2,404 (84.98)	0.85 ± 0.357
4. The hospice care team provides bereavement support for the family after the patient's death.	2,072 (73.24)	0.73 ± 0.443
5. Home hospice care is in line with China's folk customs.	1,678 (59.31)	0.59 ± 0.491
6. For children's bereavement care, children can attend funerals and even participate in preparations.	964 (34.08)	0.34 ± 0.475
7. During the terminal stages of an illness, respiratory depression medicine is appropriate for certain treatments of severe dyspnea.	812 (28.70)	0.29 ± 0.452
8. Use of Mirabilite in Shenque acupoint application can relieve ascites.	1,481 (52.35)	0.52 ± 0.499
9. Pain threshold is lowered by fatigue or anxiety.	969 (34.25)	0.34 ± 0.475
10. Men generally reconcile their grief more quickly than women.	910 (32.17)	0.32 ± 0.467
11. Individuals who are taking opioids should also follow a bowel regime.	1,468 (51.89)	0.52 ± 0.500
12. To strengthen the construction of hospice care institutions was written into the “Healthy China 2030” strategic plan.	2,100 (74.23)	0.74 ± 0.437
13. Morphine point injections can be used to relieve cancer pain in the terminal period.	1,954 (69.07)	0.69 ± 0.462
14. The most authoritative guidelines on health care planning recommend that hospice care should be provided by (1) a multi-professional hospice care team that includes the family's general physicians, (2) general physicians, (3) a multi-professional hospital team led by a pain therapist, (4) specialized nursing staff in collaboration with an anesthetist, and (5) specialized nursing staff	2,557 (90.39)	0.90 ± 0.295
15. The purposes of melodic therapy are not (1) to relieve physical pain, (2) entertainment, (3) to express emotions, (4) to evoke memories, or (5) to relieve grief	1408 (49.77)	0.50 ± 0.500
**Total**	23727 (55.91)	8.39 ± 2.780

### Attitudes

The mean score on the attitudes scale was 84.79 ± 10.561, and the total scoring rate was 70.66%. Table 3 presents the attitudes scale items and their mean scores. The mean scores of perceived susceptibility, perceived benefits, perceived barriers, and subjective norms were 2.88 ± 1.193, 4.15 ± 0.876, 2.71 ± 1.147, and 3.83 ± 0.960, respectively.

**Table 3:** Respondents' attitudes toward palliative care (*N* = 2,829).

**Items**	**Mean ±SD**
**Perceived susceptibility:**	2.88 ± 1.193
1. Uncomfortable taking care of advanced cancer patients.	2.43 ± 1.200
2. Hopeless for the cure.	3.23 ± 1.166
3. Unable to easily face the dying process and distress.	2.80 ± 1.167
4. Makes me feel weak.	2.91 ± 1.231
5. I feel guilty when an amine patient dies.	3.04 ± 1.200
**Perceived benefits:**	4.15 ± 0.876
6. Able to promote life quality and keep dignity.	4.32 ± 0.901
7. Able to die peacefully and have a good death.	4.33 ± 0.849
8. Having care and being accompanied by a medical team.	4.39 ± 0.793
9. Emotional support.	4.30 ± 0.820
10. Able to have family support.	4.22 ± 0.851
11. Respect for the patient's religion and burial rites.	4.29 ± 0.871
12. Help to die at home.	3.59 ± 1.004
13. Better communication with advanced patients.	4.14 ± 0.857
14. Help medical staff to take care of patients better.	4.23 ± 0.826
15. Avoid the idea of euthanasia.	3.65 ± 0.987
**Perceived barriers:**	2.71 ± 1.147
16. Shorten a patient's life, just like euthanasia.	2.32 ± 1.175
17. No active treatment for physical symptoms.	2.67 ± 1.165
18. Make patients feel hopeless.	2.30 ± 1.145
19. Advanced patients have many complex symptoms.	3.45 ± 1.065
20. Keep providing long-term hospice care services will lose enthusiasm.	2.83 ± 1.183
**Subjective norms:**	3.83 ± 0.960
21. It is meaningful.	4.17 ± 0.877
22. I experienced the death of my family member, which affected me to provide hospice care.	3.50 ± 1.060
23. It is a part of the duty of medical staff.	3.96 ± 0.929
24. With the approval and support of the department leader, colleagues, relatives, and friends, I was encouraged to provide hospice care.	3.70 ± 0.975
**Total**	84.79 ± 10.561

### Self-efficacy and practices

The mean score of self-efficacy was 40.59±7.691, which was approximately 73.80% of the total score. The mean score of practices was 46.42±11.959, which was approximately 66.31% of the total score. Table 4 presents the mean scores of each item.

**Table 4:** Scores of respondents' self-efficacy and practices in palliative care (*N* = 2,829).

**Items**	**Self-efficacy Mean ±SD**	**Practices Mean ±SD**
1. Alleviate the pain and discomfort of dying patients.	3.56 ± 0.946	3.54 ± 1.009
2. Make pain assessments of patients.	3.82 ± 0.873	3.54 ± 1.072
3. Reduce unnecessary treatment costs.	3.53 ± 0.917	3.26 ± 1.062
4. Satisfy the physical and mental needs of dying patients.	3.67 ± 0.940	3.55 ± 1.015
5. Explain the expected dying process to the patient's family.	3.60 ± 0.950	3.20 ± 1.086
6. Tell family specific things they can do to provide meaningful service to patients.	3.85 ± 0.836	3.43 ± 1.026
7. Understand the wishes and pain of the family to help them.	3.83 ± 0.855	3.46 ± 1.012
8. Create a good relationship between the medical staff and family members.	3.86 ± 0.871	3.65 ± 0.997
9. Coordinate the media resources for medical, social, psychological, and spiritual care.	3.64 ± 0.941	3.27 ± 1.113
10. Help risk grieving families to get through better.	3.59 ± 0.976	3.31 ± 1.053
11. Guide the management of afterward and funeral preparation for families.	3.67 ± 0.951	3.17 ± 1.129
12. Proactively talk to patients and families about death-related topics*	NA	2.99 ± 0.968
13. Proactively recommend medical institutions for end-of-life care to terminal patients and their families^*^	NA	2.84 ± 1.067
14. Talk to the patient's family proactively about “respecting the patient's wishes”^*^	NA	3.23 ± 1.044
**Total**	40.59 ± 7.691	46.42 ± 11.959
**Average**	3.69 ± 0.914	3.32 ± 1.047

^*^This entry is a question closely related to behavior and is not included in the original KAPHC scale.

NA, not available.

### Bivariate analysis

Bivariate correlations are shown in Table 5. Knowledge was positively associated with perceived benefits (r = 0.342, *P* < 0.01), subjective norm (r = 0.332, *P* < 0.01), self-efficacy (r = 0.318, *P* < 0.01), and practices (r = 0.279, *P* < 0.01). Perceived susceptibility was positively associated with barriers (r = 0.338, *P* < 0.01). Benefits were positively associated with the subjective norm (r = 0.601, *P* < 0.01), self-efficacy (r = 0.506, *P* < 0.01), and practices (r = 0.346, *P* < 0.01). Subjective norm was moderately positively associated with self-efficacy (r = 0.594, *P* < 0.01) and practices (r = 0.446, *P* < 0.01). Self-efficacy was positively associated with practices (r = 0.622, *P* < 0.01).

**Table 5:** Descriptive statistics and correlation coefficients among variables (*N* = 2,829).

	**Intention**	**Knowledge**	**Perceived susceptibility**	**Perceived benefits**	**Perceived barriers**	**Subjective norm**	**Self-efficacy**
Knowledge	0.147^**^						
Perceived susceptibility	−0.274^**^	−0.100^**^					
Perceived Benefits	0.110^**^	0.342^**^	−0.004				
Perceived Barriers	−0.196^**^	−0.092^**^	0.338^**^	−0.074^**^			
Subjective norm	0.272^**^	0.332^**^	−0.127^**^	0.601^**^	−0.086^**^		
Self-efficacy	0.298^**^	0.318^**^	−0.212^**^	0.506^**^	−0.174^**^	0.594^**^	
Practices	0.335^**^	0.279^**^	−0.197^**^	0.346^**^	−0.133^**^	0.446^**^	0.622^**^

^**^P < 0.01.

### Structural equation modeling

**Figure 2:**
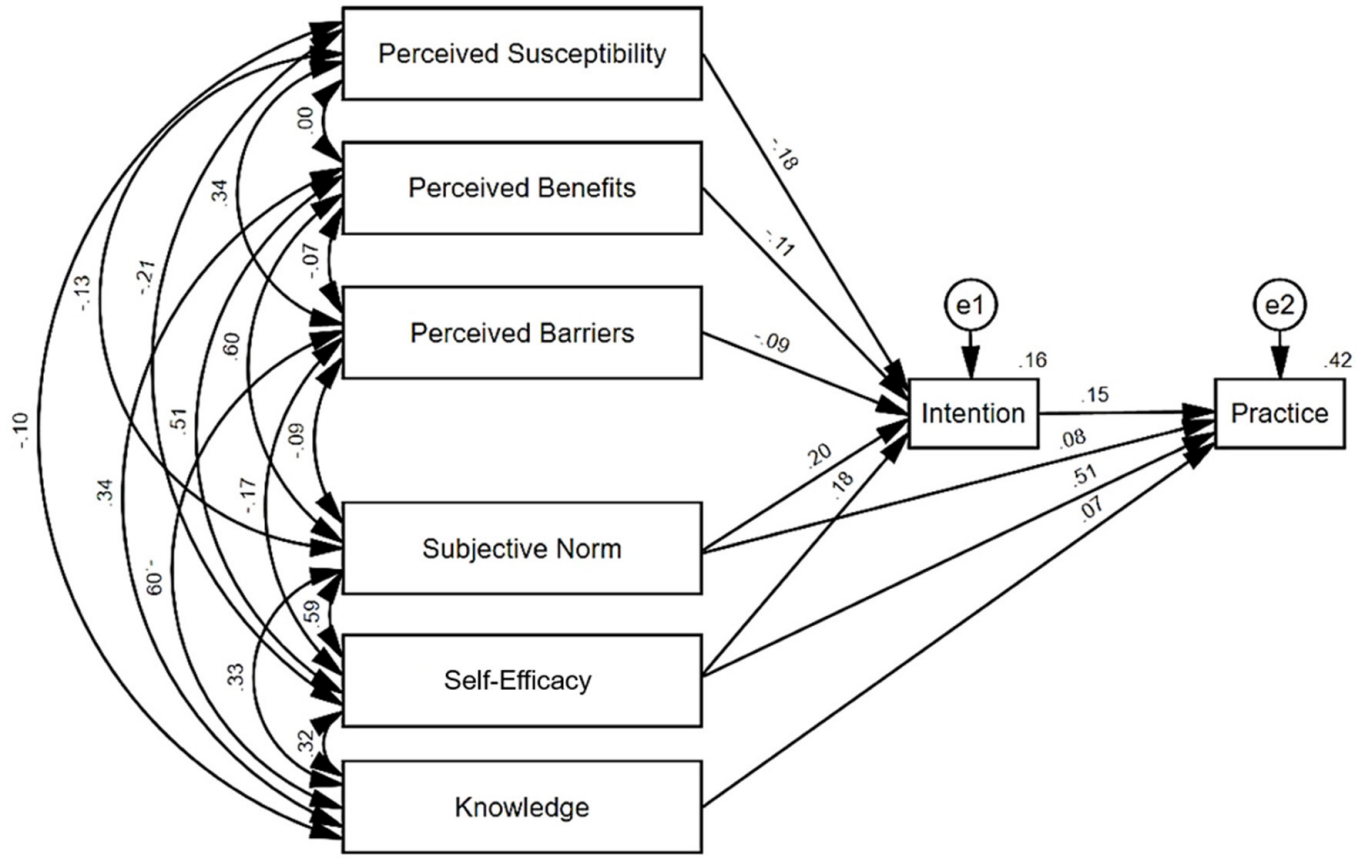
Structural equation model of nurses' practices in palliative care (standardized estimates).

The model was modified by removing the non-significant paths using AMOS until the final model showed a good model fit. The revised model is shown in Figure 2. The final model manifested a satisfactory model fit (χ^2^/df = 2.624; GFI = 0.999, AGFI = 0.992, NFI = 0.998, IFI = 0.999, TLI = 0.992, CFI = 0.999; RMSEA = 0.024, 90%CI = 0.006–0.042). The standardized direct, indirect, and total coefficients are summarized in Table 6. Several important results of this study are as follows: (1) Intention directly influenced practices. The standardized path coefficient of the direct effect is 0.152 (*P* < 0.01). (2) Subjective norm and self-efficacy had direct and indirect impacts on practices through intention. The standardized path coefficient of the direct, indirect, and total effect of subjective norm on practices was 0.082 (*P* < 0.01), 0.030 (*P* < 0.01), and 0.112 (*P* < 0.01), respectively. The standardized path coefficient of the direct, indirect, and total effect of self-efficacy on practices was 0.506 (*P* < 0.01), 0.028 (*P* < 0.01), and 0.534 (*P* < 0.01), respectively. (3) Knowledge directly influenced practices. The standardized path coefficient of the direct effect is 0.068 (*P* < 0.01). (4) Perceived susceptibility, perceived benefits, and perceived barriers had indirect effects on practices through intention. The standardized path coefficients of indirect effect are −0.027 (*P* < 0.01), −0.017 (*P* < 0.01), and −0.014 (*P* < 0.01), respectively.

**Table 6:** Direct, indirect, and total effects of variables in the final model (*N* = 2,829).

**Endogenous variables**	**Predicting variables**	**Standardized direct effect**			**Standardized indirect effect**			**Standardized total effect**		
		β	**Lower bounds**	**Upper bounds**	β	**Lower bounds**	**Upper bounds**	β	**Lower bounds**	**Upper bounds**
Intention	Perceived susceptibility	−0.179^**^	−0.216	−0.144				−0.179^**^	−0.216	−0.144
	Perceived benefits	−0.109^**^	−0.152	−0.067				−0.109^**^	−0.152	−0.067
	Perceived barriers	−0.094^**^	−0.131	−0.059				−0.094^**^	−0.131	0.059
	Subjective norm	0.199^**^	0.153	0.244				0.199^**^	0.153	0.244
	Self-efficacy	0.181^**^	0.135	0.223				0.181^**^	0.135	0.223
Practices	Perceived susceptibility				−0.027^**^	−0.036	−0.020	−0.027^**^	−0.036	−0.020
	Perceived benefits				−0.017^**^	−0.025	−0.010	−0.017^**^	−0.025	−0.010
	Perceived barriers				−0.014^**^	−0.021	−0.009	−0.014^**^	−0.021	−0.009
	Subjective norm	0.082^**^	0.042	0.118	0.030^**^	0.022	0.041	0.112^**^	0.071	0.148
	Self-efficacy	0.506^**^	0.469	0.543	0.028^**^	0.020	0.037	0.534^**^	0.498	0.570
	Knowledge	0.068^**^	0.038	0.099				0.068^**^	0.038	0.099
	Intention	0.152^**^	0.122	0.184				0.152^**^	0.122	0.184

β, standardized regression coefficient.

^**^P < 0.01.

## Discussion

Our original study has analyzed the interrelationship and intensity between practices and related factors among palliative care nurses in Shanghai, providing evidence of the associations of knowledge, perceived susceptibility, benefits, barriers, subjective norm, self-efficacy, intention, and practices aiming at palliative care nurses and interventions improving their actual work practices. The structural equation model authenticated the interrelationship and intensity between all dimensions, highlighting that self-efficacy, intention, and subjective norms greatly influenced practices among nurses concerning palliative care. Concretely, self-efficacy and subjective norms had direct and indirect impacts on practices. Intention and knowledge directly influenced practices. Perceived susceptibility, perceived benefits, and perceived barriers had indirect effects on practices through intention. The above hypotheses are verified by the model.

Our study found that the average score rating of nurses' practices (66.3%) was lower than that of all health providers in Shanghai (74.5%) (19) and that of oncology nurses in Shandong Province (82.2%) (20). This result may be in part because the majority of the participants in our study worked in primary healthcare institutions, including community health centers and nursing homes. In the initial stage of palliative care pilot work in Shanghai, there is a shortage of multidisciplinary team composition, and nurses undertake multi-functional roles and diversified nursing in primary healthcare institutions. Strategies to improve nurses' practices should be addressed when developing interventions.

In the model, self-efficacy, which was defined as the degree of confidence of nurses in the provision of palliative care services in this study, had profound the most important direct impacts on practices, indicating that nurses with better confidence have a stronger disposition to practices. A cross-sectional study showed that exploring the traditional Chinese philosophy of life was essential for the improvement of hospice care self-efficacy (21). Another study showed a positive and statistically significant correlation between communication skills and self-efficacy (22). Therefore, it is crucial to strengthen communication skills and enhance confidence through comprehensive and systematic training in respect of Chinese traditional concepts of life and death in order to further promote practical palliative care practice. Meanwhile, this result was in line with the previous study in which a significantly positive association between self-efficacy and practices has been observed (23), indicating that it is also a critical predictor and appropriate index measuring the self-efficacy of nurses to evaluate the level of practices.

Intention, which was defined as a willingness to practice, directly influenced and had a significantly positive association with practices. As shown in the model, the intention was positively affected by subjective norms and self-efficacy and negatively affected by perceived susceptibility to the condition of terminal patients' deterioration and perceived barriers to palliative care service provision. An analysis showed that whether nurses had clinical hospice care experience affected their willingness to practice (23). However, the demographic characteristics of the respondents showed that a part of the participants had no experience with death witnesses and was unwilling to provide palliative care services, which reduced practice frequency. Therefore, nurses should be emboldened to improve their behavioral intention by participating in the real case of end-of-life, improving clinical practices and training on death, and establishing an objective view of life and death.

Subjective norms also had direct and indirect impacts on practices, indicating that social values, expectations of leaders, and encouragement of colleagues had a great impact on the provision of palliative care services. There is a significantly positive correlation between subjective norms and practices, which was in line with another study (24), manifesting that the potential usefulness of subject consciousness and motivation in respect of palliative care services were self-evident. Strategies enhancing norm belief and compliance motivation and interventions, such as implicit messages and intentional teaching, should be considered (25).

The model also revealed the direct effects of knowledge on practices, reflecting the effectiveness of continuing education to improve nurses' competence in providing qualified palliative care services. Empirical research proved that targeted training in palliative care can address the knowledge and skills gaps in time. Moreover, a significantly positive correlation between knowledge and practices was obtained, which resembled another study (10). Despite the frequent phenomenon of knowing without doing, the great majority of researchers hold the view that the effect of knowledge on practices is worth affirming fully and permanently among other factors (26). Previous study showed that the lack of adequate end-of-life and legal literacy training for nurses was a key reason why the overall knowledge of health service providers in Shanghai was generally moderate (21) and a lack of adequate training on end-of-life will and legal knowledge for nurses is a key reason (27). Consequently, it is highly necessary for nurses, the major primary health service providers in diversified nursing, to systematically learn theoretical knowledge in palliative care, strengthen education on life and death, and improve the understanding of the concept and significance of palliative care services.

Perceived susceptibility, benefits, and barriers had negative indirect effects on practices through intention. First, perceived susceptibility had the most dramatic effect among three dimensions, indicating that nurses always felt upset, anxious, weak, hopeless, guilty, suffocated, and grieved and were unable to easily face the dying process of coping with patient death, which was unanimous with another study (28). Second, perceived barriers had indirect effects on practices, showing that nurses who had more perceived difficulties concerning palliative care had lower behavior, which was consistent with a previous study (29). Finally, perceived benefits had an indirect effect on practices. This phenomenon manifests that although nurses can realize the benefit of patients receiving palliative care is that patients are able to die peacefully and had a good death with omnidirectional care, highlife quality, and dignity (30). The patient's religion and burial rites should be respected (31). However, some participants perceived the provision of palliative care services as a serious burden, concerning occupational prospects, low salaries, and coping with a broad range of stressors in actual work practices. Thus, perceived susceptibility and barriers remain dominant factors, which is showing no difference with another study (32). Hence, it is necessary to provide effective strategies to treat themselves with empathy, kindness, and awareness of common humanity in clinical practices, contributing to their wellbeing.

Knowledge and attitudes are the antecedents and prerequisites. Before producing behavior, people transform external and internal needs into motivation and purpose through self-consciousness and understanding, to guide and regulate behavior and practices. After the change in knowledge and attitudes, behavior ultimately changes. A previous study showed that, in regular and continuous behavior, there is a two-way influence in the relationship between motivation, attitudes, beliefs, and behavior (33). In providing palliative care services, nurses' work practices will, in turn, promote changes in their own knowledge, attitudes, subjective norms, self-efficacy, and intention.

### Strengths and limitations

The availability and accessibility of palliative care continue to be major global public health problems and are more challenging in China. However, few studies have investigated the interrelationship and intensity between practices and their influencing factors among nurses in palliative care. To our best knowledge, this is an original large-scale empirical study that has meticulously analyzed the interrelationship and intensity between practices and other factors among nurses in the initial stage concerning palliative care in primary healthcare institutions in Shanghai, China. Our findings have some practical implications and valuable information for nurses concerning palliative care. High-quality palliative care services can be achieved only by scaling up interventions to enhance self-efficacy, intention, subjective norm, and knowledge to motivate nurses to provide patient-centered integrated, and comprehensive behavior.

However, several limitations should be noted. First, most recent health behavior theories used in China originated from western culture. Because behavior is the profound embodiment of culture, the suitability of these theories in China is worth considering. Furthermore, most of the participants worked mainly in primary healthcare institutions. There is an urgent need for future basic and applied research studies on the influencing factors on nurses' practices in secondary and tertiary medical institutions. Finally, although the structural equation model is an advanced and reliable quantitative analysis method, it still contains subjective cognition from researchers. Therefore, other several complex mathematical models and analysis methods should be considered in further study to avoid bias.

## Conclusion

This study preliminarily established a theoretical foundation by structural equation model among 2,892 nurses in healthcare institutions in Shanghai. The hypothetical model verified the interrelationship and intensity between nurses' practices of palliative care and several significant factors; Intention and knowledge directly influence practices; Subjective norm and self-efficacy have both direct and indirect impacts on practices through intention; Additionally, perceived susceptibility, perceived benefits, and perceived barriers had indirect effects on practices through intention. Ultimately, it is imperative to scale up targeted interventions focusing on self-efficacy, intention, and subjective norms to improve the practices of nurses, especially for nurses working in primary healthcare institutions. High-quality palliative care services can be achieved by motivated nurses with strong practical nursing abilities.

## Data availability statement

The raw data supporting the conclusions of this article will be made available by the authors, without undue reservation.

## Ethics statement

Written informed consent was obtained from the individual(s) for the publication of any potentially identifiable images or data included in this article.

## Author contributions

LJ secured the funding to conduct this research and was responsible for conceptualizing the manuscript. JH participated in project design and grant article publication. YX and XT collected and analyzed the data. ZS contributed to the design of the scale. YX and SZ drafted the initial manuscript. ML and TC revised and polished the manuscript. JW and YM proofread the logical framework. SL coordinated the investigation. All authors were responsible for critical revisions and approval of the final manuscript.
